# Report of Deaths in Buffalo for the Month of August, 1855

**Published:** 1855-10

**Authors:** J. Root

**Affiliations:** Health Physician


					﻿ART. IX.—Report of Deaths in Buffalo for the month
AGE.
"O O O £
K ■*J a
O _ -1
DISEASES.	-----------
. S
cc "rt ,—<■	® «
~ S -S a> g « g
*	S	Ph	H	®	>5	A1
S S A
Accident,............................................. 2	1	3	....	1	..
Apoplexy,............................................. 3	1	4	1......
Anaemia,.................................................... 2	2 ..........
Atrophia,................................................... 1	1 .. 1....
Consumption, ........................................	8	13	21	3	2	..--
Cholera,.............................................. 5	1	6	.........
“ Infantum,..................................... 20	8	28	14	3	3	6
“ Morbus,........................................ 1	1	2	...........
Convulsions,.......................................... 13	7	20	8	3	3	2
Chronic Gastritis,.................................... 1	....	1	.........
Cancer,............................................... 1	....	1	 .....
Child Bed,.................................................. 1	1	.........
Congestion of Brain,.................................. 2	....	2	....	1..
Croup,....................................................   2	2	.........
Diarrhoea (summer complaint),........................ 47	29	76	<5	1611 10
Debility.............................................. 6	3	9	5	31..--
Dysentery,............................................ 7	6	13	4412
Drowned,.............................................. 8	....	8	......
Dentition,............................................ 4	4	8	3	2 1 2
Dropsy,............................................... 4	1	5 ..........
Disease of Brain,........................................... 1	1	.........
Delirium Tremens,..................................... 1	....	1	.........
Epilepsy,................................................... 1	1	.........
Fever,................................................ 1	....	1	.........
“ Scarlet,......................................... 2	2	4	....... 2
“ Typhoid,....................................... -—	3	3 .......
“ Typhus,.......................................... 1	1 2 ............
Fracture of Spine,.................................... 1	....	1	.........
Hydrocephalus,........................................ 2	4 6 1.. 1 3
Insanity,............................................. 1	....	1	.........
Inanition,......................................,..... 1	.—	1	1........
Inflammation of Bowels,....................................  2	2	......... 1
Inflammation of Brain,................................ 1	-.--	1	........
Measles,.............................................. 3	2	5	1	....	1
Marasmus,............................................. 2	2	4	1	2 1	- -
Neuralgia,............................................ 1	-—	1	........
Old Age,.............................................. 1	1	2	........
Obstruction of Bowels,................................ 1	-—	1	........-■
Premature Birth,...................................... 1	1	2	1	1 ..	- -
Paralysis............................................. 1	-—	1	........
Puerperal Convulsions,..............................-—	1	1	.........
Pyemia,.............................................-—	1	1	.........
Still-born,........................................... 6	3	8	5 3 ....
Spina Bifida,.......................................----	1	1	1........
Scrofula, ....t.....................................----	1	1	.........
Suicide by Laudanum,.................................. 1	----	1	.........
Softening of Brain,................................... 1	....	1	.........
Typhoid Pneumonia,..................................----	1	1	.........
Unknown,............................................. 20	11	31	11	9 1 ..
Whooping Cough,....................................... 2	2	4	1	2 1 - -
Totals,...........................T182~	122~ 304~ 96 5? 25 29
i	Nativities.—American, 128. German, 82. English, 6. Irish, 20. French, 7.
of August, 1855. By J. Root, M. D., Health Physician.
AGE.
in o »-«Qic*cQ'^<in<oi^cocnc>5h.£
I7llllllllll7o^“
p
qj®aja5a/®®ffll®®a)fflQ® ®
ai S S o 2 cj 2 «j 2 ® 2 a> ? ,2 - ® g ® 2 ® 2 ® 2 «5 2 a § <□ 2
42 5 42 5 =• ® 43 5 rt ® S 42 ® <S 3 ® — > 42 ® 42 S ” « ~ ® 42 ®
....................... 1 .. ..!.. .. 1...........
1........ 1.......... 1....I....................
.............. 1	-	- 1 . . I.
.................................I.
1	■’ ’’ i " i ’’ ’’ i"‘2*i*3-- 3 i i i
........... i .. 1.. i.-- i i'.. i..............................
2	.........................I...................-
........... 1 i ................................................
2 2........................!........
..............
................... 1.............................
	 i	-...:.......................
.. 2............................................................
4.................................................
............................... 1.............
................................ 1 .. 1.,.......................
1	.......... 2 -- 3 .. 2..........................
_ _ _..
” ■'" i " ” " *i'.'. " '.'. '.'.'.'." ’’ i *i '■ " " ~ i " " "	'.'.
...................... 1......................
...................................................................... 1...................................
........................................................ 1.
....................... 1.........................
2	............!-................................
... 1......... 1.. .. .. 1........................
.. 1..........i .. .. 1...........................
‘‘ " ” ” " ” " . " " .. ■’ 1 " " " ” " " " " " ’■ .. " "
■■ ’■" ’■" " :: ■’:: ■■ ” " " i*."" ■ ::":: ” ""
i .J............................................
i 11............................................
............................. ........
............................................................................i.....................................
............................................................................i.i.
.................................................... i.
................ i .. /... .. ............ ’’ ’’..
.................. 1................
.................... .. i .......................
’’ ’’.... "..’’..’’.. 7. " .. ’’ i.... ’’ ’’ ” ’’'' ’’ ’’ ’’ ’’..
............................................................................................ 1..
..............................................i............................................. i.
.............................................. i.
1 1 2 .. 1.... 1 2..... 1..... 1................
i6“7'3_2l_0_3^’81_5_7~4 “6_8"8’7_3_2_5_3 “olll’ITOl’b
Scotch, 3. Hollander, 6. Prussian, 2. Swiss, 2. Country not given, 44.—Total, 304’
				

